# Nisin S, a Novel Nisin Variant Produced by *Ligilactobacillus salivarius* P1CEA3

**DOI:** 10.3390/ijms24076813

**Published:** 2023-04-06

**Authors:** Ester Sevillano, Nuria Peña, Irene Lafuente, Luis M. Cintas, Estefanía Muñoz-Atienza, Pablo E. Hernández, Juan Borrero

**Affiliations:** Departamento de Nutrición y Ciencia de los Alimentos (NUTRYCIAL), Sección Departamental de Nutrición y Ciencia de los Alimentos (SD-NUTRYCIAL), Facultad de Veterinaria, Universidad Complutense de Madrid (UCM), Avenida Puerta de Hierro, s/n, 28040 Madrid, Spain

**Keywords:** antimicrobial peptides, nisin, lantibiotic, *Ligilactobacillus salivarius*, bacteriocin

## Abstract

Recently, the food industry and the animal farming field have been working on different strategies to reduce the use of antibiotics in animal production. The use of probiotic producers of antimicrobial peptides (bacteriocins) is considered to be a potential solution to control bacterial infections and to reduce the use of antibiotics in animal production. In this study, *Ligilactobacillus salivarius* P1CEA3, isolated from the gastrointestinal tract (GIT) of pigs, was selected for its antagonistic activity against Gram-positive pathogens of relevance in swine production. Whole genome sequencing (WGS) of *L. salivarius* P1ACE3 revealed the existence of two gene clusters involved in bacteriocin production, one with genes encoding the class II bacteriocins salivaricin B (SalB) and Abp118, and a second cluster encoding a putative nisin variant. Colony MALDI-TOF MS determinations and a targeted proteomics combined with massive peptide analysis (LC-MS/MS) of the antimicrobial peptides encoded by *L. salivarius* P1CEA3 confirmed the production of a 3347 Da novel nisin variant, termed nisin S, but not the production of the bacteriocins SalB and Abp118, in the supernatants of the producer strain. This is the first report of a nisin variant encoded and produced by *L. salivarius*, a bacterial species specially recognized for its safety and probiotic potential.

## 1. Introduction

Antibiotics have been widely used in swine production as prophylactic or therapeutic agents to combat pathogenic bacteria. The extensive use of antibiotics and the continuous exposure of bacteria to non-lethal doses of these compounds have accelerated the emergence of multidrug-resistant bacteria [1], which is now considered to be one of the greatest threats to human health worldwide [2]. To control this potential health crisis, since 2022, the European Union (EU) has introduced new laws regarding the use of antibiotics in farming, ending all forms of routine antibiotic use and, in particular, their use to compensate for inadequate husbandry and poor hygiene [3]. Accordingly, there is an urgent need for a replacement to the use of antibiotics in animal production, and different strategies are being considered, including the use of antimicrobial peptides such as bacteriocins. Bacteriocins are non-toxic peptides produced by bacteria, with antimicrobial activity against other bacteria, including pathogenic and/or antibiotic-resistant strains [1]. Bacteriocin production is widespread among Gram-positive and Gram-negative bacteria, and it is considered, in many cases, to be a probiotic trait indicating that these peptides exert a regulatory role in the bacterial ecosystems, including the gut microbiome [4]. Moreover, bacteriocin production and secretion to the extracellular environment by beneficial gut bacteria represent a significant advantage over conventional drug administration, as some of these bacteriocins can be produced in situ and secreted to the gastrointestinal tract (GIT), thus controlling intestinal infections [5]. Several studies have already shown the beneficial effects of using bacteriocin-producing probiotics as a promising strategy for the treatment of gastrointestinal disorders in animals [6].

Bacteriocins produced by Gram-positive bacteria can be divided into two main classes: class I or lantibiotics with modified amino acid residues, and class II bacteriocins with unmodified amino acid residues. Lantibiotics are post-translationally and enzymatically modified peptides with lanthionine and β-methyl-lanthionine residues produced by the dehydration of serine (into dehydroalanine) and threonine (into dehydrobutyrine), which form lanthionine bridges when reacting with cysteine thiols [7,8]. Most lantibiotics are able to bind the membrane-bound peptidoglycan precursor lipid II and use it as a docking molecule, killing the target strain by two different strategies: formation of pores in the cytoplasmic membrane and inhibition of the cell wall synthesis [8]. Most likely, this dual mode of action is one of the causes for the low levels of naturally occurring lantibiotic resistance observed [9,10]. Lantibiotics can be further subdivided into three classes: class Ia (linear lantibiotics), class Ib (globular lantibiotics), and class Ic (multicomponent lantibiotics) [1,11]. Nisin A, produced by *Lactococcus lactis*, is the prototypical lantibiotic from class Ia and the only bacteriocin licensed as a biopreservative [12,13]. Nisin has been extensively used in the food industry to control the growth of foodborne pathogenic bacteria such as clostridia or *Listeria* strains, among others [7,14]. However, the application of nisin as an antimicrobial agent has recently been extended to other fields such as human and animal health.

Since its discovery, twelve natural variants of nisin (nisin A, Z, Q, U, U2, F, H, O, J, P, G, and E) [8,12,15,16,17,18,19,20,21,22,23] and two nisin-like bacteriocins (salivaricin D and kunkecin A) [24,25] have been characterized. Natural nisin variants are between 31 and 35 amino acid long and have been identified as produced by the bacterial species *Lactococcus lactis* (nisin A, Z, F, and Q), *Streptococcus* sp. (nisin P, U, U2, J, H, G and E, and salivaricin D), *Blautia obeum* (nisin O_4_ and O_1,2,3_), and *Apilactobacillus kunkeei* (kunkecin A). It has also been observed that nisin variants originating from the same or closely related species exhibit greater similarity to each other than to variants from other genera. Despite the differences in their amino acid sequences, all nisin variants share a similar three-dimensional structure consisting of five lanthionine rings (A, B, C, D, and E) distributed in two well defined domains: an amphiphilic N-terminal region involved in the recognition and binding to lipid II and a C-terminal region involved in the pore-forming activity. The domains are connected together by a flexible hinge region [26,27]. A number of nisin-derived synthetic variants have been bioengineered with, in some cases, enhanced bioactivity and/or spectrum of activity regarding their natural counterparts [27,28,29,30].

In this study we identified a novel nisin variant produced by *Ligilactobacillus salivarius* P1CEA3, isolated from the GIT of pigs. Whole genome sequencing (WGS), colony Matrix-Assisted Laser Desorption/Ionization Time-of-Flight Mass Spectrometry (MALDI-TOF MS) determinations, and purification to homogeneity of the secreted antimicrobial peptides confirmed the production by *L. salivarius* P1ACE3 of a novel nisin variant, termed nisin S (NisS). This bacteriocin shows a high antimicrobial activity against pathogens of interest in swine production including *Streptococcus suis*, *Enterococcus faecalis*, and *Clostridium perfringens*. This is the first nisin variant produced by *L. salivarius*, a lactic acid bacteria (LAB), with the status of qualified presumption of safety (QPS) by the European Food Safety Authority (EFSA) [31] and a LAB species that has gained attention as a promising probiotic bacteria [32].

## 2. Results

### 2.1. Direct and Extracellular Antimicrobial Activity of L. salivarius P1CEA3 and Stability to pH, Heat, and Proteases

*L. salivarius* P1CEA3, isolated from the GIT of pigs and identified at the species level, was selected due to its high direct antimicrobial activity against *Streptococcus suis* and other Gram-positive pathogens of interest in swine production (Table 1).

To optimize the growth of *L. salivarius* P1CEA3 and to enhance the production of antimicrobial compounds in the cell-free supernatants (CFS), various growth conditions were tested such as different culture media and bacterial growth in both aerobic and anaerobic environments. The final OD_600_ of *L. salivarius* P1CEA3 cultures varied depending on the medium used, with the highest OD_600_ obtained in the MRS medium after 16 h of growth in anaerobic conditions. Notably, cultures grown in TSB exhibited the highest specific antimicrobial activity (SAA) in their CFS, followed by cultures grown in BHI and MRS. Furthermore, the highest SAA in the supernatants of *L. salivarius* P1CEA3 was observed after 72 h of anaerobic growth in 1 L of TSB (Table S1). The antimicrobial activity in the CFS of *L. salivarius* P1CEA3 remained stable after 37, 70, 100, and 120 °C treatments for 10 min. However, variations in antimicrobial activity were observed under different pH conditions, with the highest activity observed under acidic conditions (pH from 2.0 to 4.5). Further, treatment with proteinase K led to the total loss of antimicrobial activity, confirming the proteinaceous nature of the antimicrobial compounds present in the CFS of *L. salivarius* P1CEA3.

### 2.2. Identification of Two Bacteriocin Gene Clusters in the L. salivarius P1CEA3 Genome

**Figure 1:**
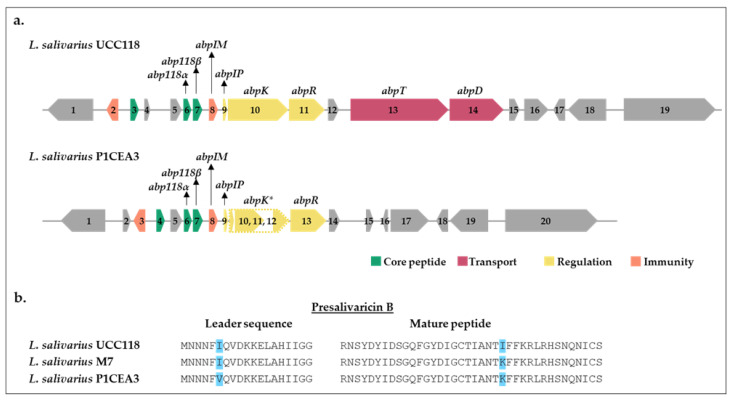
Comparison of the gene clusters from *L. salivarius* UCC118 and *L. salivarius* P1CEA3. ORFs are indicated by numbers and arrows, and those with a known function are indicated by gene identity and color. (**a**) ORFs 10–12 (so called abpK*) code for a truncated version of AbpK from *L. salivarius* UCC118; (**b**) comparative alignment of the amino acid sequences of presalivaricin B in *L. salivarius* UCC118, *L. salivarius* M7, and *L. salivarius* P1CEA3. Amino acid differences are highlighted in blue.

WGS of *L. salivarius* P1CEA3 (GenBank accession number CP116812-CP116815) and the subsequent analysis of the assembled sequences with the bacteriocin mining tool BAGEL4 revealed the presence of two different bacteriocin gene clusters. One cluster encodes bacteriocins identified as salivaricin B (SalB) and Abp118 (Abp118α + Abp118β), both previously found clustered together in *L. salivarius* UCC118 [33] (Figure 1a). Notably, there are two amino acid differences between ORF3 (presalivaricin B) in *L. salivarius* UCC118 and SalB found in *L. salivarius* P1CEA3, one located in the leader sequence (I6V) and the other in the mature peptide (I24K). However, mature SalB in *L. salivarius* P1CEA3 holds the same amino acid sequence as the salivaricin B (SalB) encoded by *L. salivarius* M7 [34] (Figure 1b).

In addition, the *abp118* gene cluster in *L. salivarius* P1CEA3 shows a partial homology with that in *L. salivarius* UCC118 [33] (Figure 1a). Although the structural genes, *abp118α* and *abp118β*, present one and two nucleotide substitutions, respectively, compared to those in *L. salivarius* UCC118, the amino acid sequences of both peptides are identical. The peptides and proteins encoded by *abpIM*, *abpIP,* and *abpR* in *L. salivarius* P1CEA3 have a 89%, 100%, and 97% identity, respectively, with the immunity protein AbpIM, the induction factor AbpIP, and the response regulator AbpR produced by *L. salivarius* UCC118 [33] (Table S2). However, the ORFs 10–12 from *L. salivarius* P1ACE3 have a certain homology with *abpK* which encodes the histidine kinase protein (AbpK) in *L. salivarius* UCC118. The abpK* sequence in *L. salivarius* P1CEA3 (ORFs 10–12) presents different single and multiple nucleotide deletions responsible for frameshift mutations, thus generating a truncated version of the AbpK protein in relation to that from *L. salivarius* UCC118 [33] (Table S2). Meanwhile, the *abpT* and *abpD* genes from *L. salivarius* UCC118 encoding proteins involved in the processing and transport of Abp118 are absent in *L. salivarius* P1CEA3 (Figure 1a). Thus, since AbpK, AbpT, and AbpD are involved in the synthesis, processing, and secretion of Abp118, it is expected that this bacteriocin will not be produced and secreted by *L. salivarius* P1CEA3.

**Figure 2:**
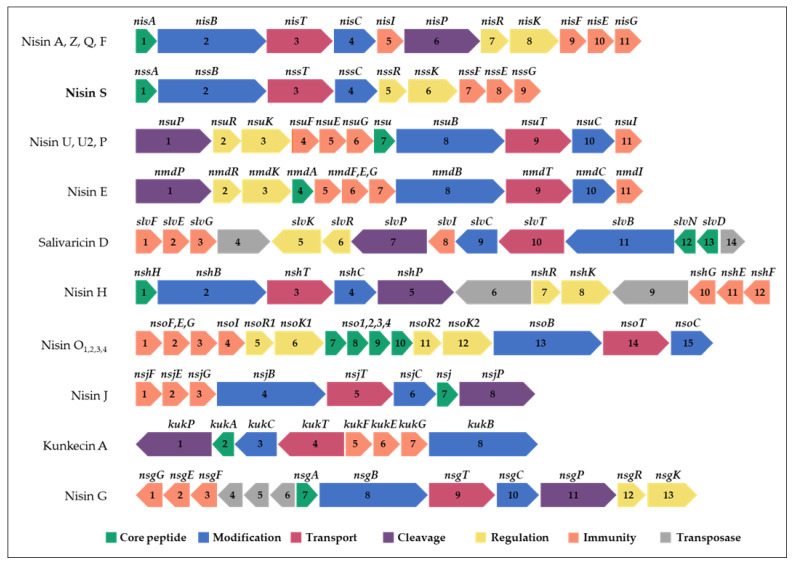
Comparison of bacteriocin gene clusters of different nisin variants. ORFs are indicated by numbers, and those with a known function are indicated by gene identification and color.

**Figure 3:**
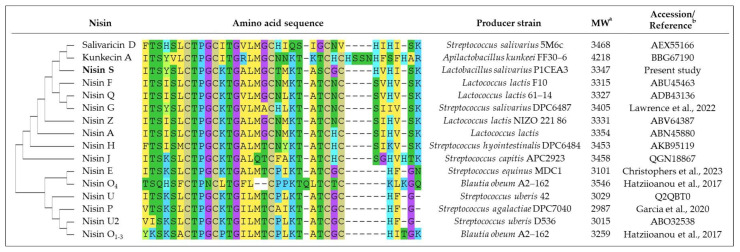
Dendrogram and multiple-sequence alignment of mature nisin variants, including NisS (in bold), observed molecular weight (MW), and accession number. For alignment and phylogeny reconstruction of the nisin variants, the MEGA11 software was used [35]. ^a^ Molecular weight (MW) of nisin variants in daltons, and ^b^ reference is given when the accession number is not available [20,21,22,23].

The second bacteriocin gene cluster found in *L. salivarius* P1CEA3 presents a set of genes presumably encoding proteins involved in the synthesis and production of a putative nisin variant, initially termed nisin S (Figure 2 and Table S3). The nisin S gene cluster in *L. salivarius* P1ACE3 is 10,385 bp long and one of the shortest among the nisin gene clusters described so far. The organization of genes within the nisin S cluster (*ABTCRKFEG*) follows the same structure as that of nisin A, but lacks the genes *nisI* and *nisP*, encoding a nisin-specific immunity protein and a leader sequence cleavage proteinase, respectively. The structural *nssA* encodes a 58-amino acid precursor with a 25-amino acid leader sequence and a cleavage site that, once processed, may result in a 34-amino acid mature peptide (Table S3). The putative mature nisin S is a 34 amino acids long peptide with a 79.41% identity with nisin A and a 82.35% identity with nisin F [12], the closest related nisin variant (Figure 3). Nisin S also contains two unique amino acids i.e., T20 and S25 compared to other natural nisin variants (Table S3).

### 2.3. In Vitro Cell-Free Protein Synthesis (IV-CFPS) and Evaluation of the Antimicrobial Activity of Abp118 (Abp118α + Abp118β), Salivaricin B (SalB), and Nisin S

**Figure 4:**
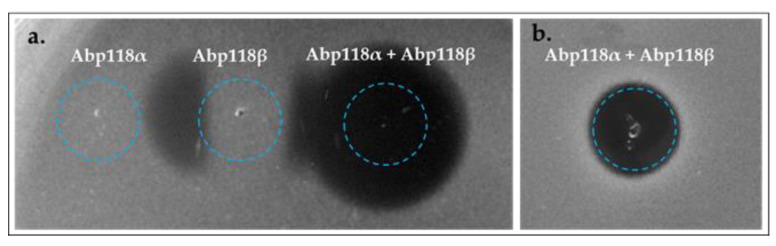
Antimicrobial activity of the IV-CFPS-produced Abp118α and Abp118β bacteriocins, alone or together, by using the spot-on-lawn test (SOLT) against *P. damnosus* CECT 4797 (**a**) and *L. salivarius* P1ACE3 (**b**), as the indicator organisms. The blue circle indicates the position and area occupied by each of the fractions tested.

To evaluate the functionality of the Abp118 (Abp118α + Abp118β) and SalB peptides, genes encoding the corresponding mature bacteriocins were initially amplified from the genomic DNA of *L. salivarius* P1CEA3, using forward and reverse primers including the T7 promoter and transcription terminator regions, respectively (Table S4). These amplicons were further used as templates for the in vitro cell-free protein synthesis (IV-CFPS) of both bacteriocins and for evaluation of their antimicrobial activity against different indicator strains. On the one hand, the in vitro synthesized Abp118α and Abp118β bacteriocins did not show antimicrobial activity when tested separately, but the combination of both peptides showed antagonistic activity against *Pediococcus damnosus* CECT 4797, *Ligilactobacillus salivarius* P1CEA3 (Figure 4), *Listeria seeligeri* CECT 917, and *Listeria monocytogenes* CECT 4032 (results not shown). On the other hand, the in vitro synthesized SalB did not show antagonistic activity against any of the indicators tested (results not shown), suggesting that either the indicators used in this study are not sensitive to SalB or that this peptide is not a functional bacteriocin.

### 2.4. Colony MALDI-TOF MS for Identification of the Nisin S Produced by L. salivarius P1CEA3

**Figure 5:**
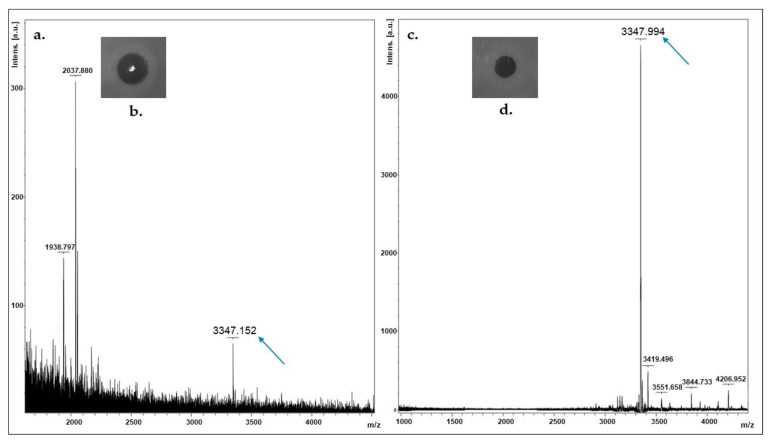
(**a**) Colony MALDI-TOF MS of *L. salivarius* P1ACE3; (**b**) direct antimicrobial activity of a single colony against *P. damnosus* 4797. (**c**) MALDI-TOF MS of the RP-FPLC active fraction from a purified supernatant of *L. salivarius* P1ACE3; (**d**) antimicrobial activity of the eluted fraction against *P. damnosus* CECT 4797.

MALDI-TOF MS of an isopropanol-derived extract from colonies of *L. salivarius* P1CEA3, grown in TSB and with antimicrobial activity against *P. damnosus* CECT 4797, revealed the presence in the extracts of a peptide of 3347.15 Da (Figure 5a,b). The 144 Da difference between the deduced (3.491 Da) and the observed (3347.15 Da) molecular mass of the identified peptide most likely corresponds to dehydration of eight amino acid residues in mature nisin S (NisS).

### 2.5. Purification of Bacteriocins from Supernatants (CFS) of L. salivarius P1ACE3, and MALDI-TOF MS Analysis of the Purified Fractions

For purification of the bacteriocins produced by *L. salivarius* P1ACE3, previously grown in TSB, the corresponding cell-free supernatants (CFS) were subjected to precipitation with ammonium sulfate, desalted by gel filtration, and further subjected to hydrophobic-interaction chromatography, followed by two rounds of reverse-phase chromatography in an ÄKTA purifier fast protein liquid chromatography (RP-FPLC) system. The MALDI-TOF MS analysis of the RP-FPLC purified fractions with antimicrobial activity showed a peptide fragment of 3347.99 Da (Figure 5c,d), sharing the same molecular mass as that observed by colony MALDI-TOF MS (Figure 5a,b). No peptide fragments of the deduced molecular mass for SalB and Abp118 (Abp118α + Abp118β) were observed.

### 2.6. Massive Peptide Analysis of Trypsinized RP-FPLC-Purified Fractions from Supernatants of L. salivarius P1ACE3, and Predicted Structure of the Nisin S

**Figure 6:**
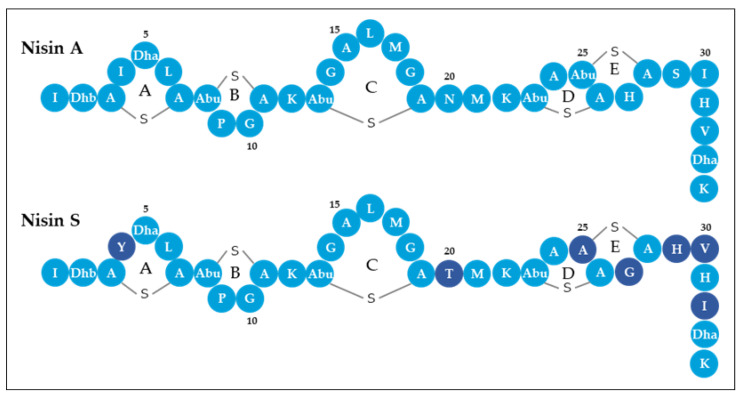
Predicted structure of the novel nisin S variant, using the deduced nisin A structure as a template. Post-translational modifications are indicated as follows: (Dha) dehydroalanine, (Dhb) dehydrobutyrine, (A-S-A) lanthionine, and (Abu-S-A) 3-methyllanthionine. Nisin rings are indicated as A, B, C, D, and E. Numbers indicate amino acid position. Differences in amino acids are shown as dark blue.

The RP-FPLC-purified fractions from supernatants of *L. salivarius* P1ACE3 with antimicrobial activity were subjected to trypsin digestion, the resulting peptide fragments were subjected to LC-MS/MS analysis and, finally, evaluated by a targeted proteomics approach combined with massive peptide analysis. Trypsinized peptide fragments from the deduced NisS were identified, covering 100% of its amino acid sequence. However, none of the deduced trypsinized peptide fragments from bacteriocins SalB and Abp118 (Abp118α + Abp118β) were identified in the purified fractions (Table 2). After detection of NisS in the supernatants of *L. salivarius* P1ACE3 and using the deduced nisin A (NisA) structure as a template, the structure of NisS was predicted as having five rings stabilized by a lanthionine bridge between S3 and C7; three methyllanthionine bridges between T8 and C11, T13 and C19, and T23 and C26; and a lanthionine bridge between S25 and C28 (Figure 6).

### 2.7. Antimicrobial Activity of Nisin S

The antimicrobial activity and spectrum of activity of the NisS, produced by *L. salivarius* P1ACE3 and purified after two rounds of RP-FLPC, were evaluated against different indicator strains. The antimicrobial activity of NisS was compared with that of NisA, purified from *L. lactis* subsp. *lactis* BB24, following the identical purification procedure as that followed for the purification of NisS. The bacteriocins were both evaluated at the same concentrations (i.e., μg/mL). The NisA, purified from lactococcal strain *L. lactis* subsp. *lactis* BB24, showed a molecular mass of 3351 Da (Figure S1), identical to that previously described for this bacteriocin (Figure 3). The purified NisS was active against most of the Gram-positive indicator strains tested and *E. coli DH5α* (Table 3). Strains that were resistant to NisS were *Corynebacterium bovis* AM3, *Bacillus cereus* ICM17/00252, and *Salmonella paratyphi* CECT 554. The NisS showed a slightly broader spectrum of activity and an overall higher antimicrobial activity as compared to that of the purified NisA.

## 3. Discussion

The massive use of antibiotics in animal farming has led to the emergence of antimicrobial resistance (AMR) in pathogenic bacteria, which is a major concern [36]. As such, bacteriocins and bacteriocinogenic bacteria hold great potential for mitigating the negative impacts of AMR in animal farming [37,38]. In an attempt to isolate potential probiotic strains adapted to the GIT of pigs, a screening of LAB with antimicrobial activity was attempted, using as source the gastrointestinal content of pigs (results not shown). One of the isolates obtained showed significant antimicrobial activity against most of the Gram-positive indicators tested (Table 1), and was identified as *Ligilactobacillus salivarius* P1CEA3.

WGS of *L. salivarius* P1CEA3 and further BAGEL4 bioinformatic analysis, revealed the presence of two different bacteriocin gene clusters. One of the clusters encodes salivaricin B (presalivaricin B from *L. salivarius* M6) and the bacteriocin Abp118 (Abp118α + Abp118β). The SalB encoded by *L. salivarius* P1ACE3 (Figure 1b) differs in one amino acid (V6I) with the presalivaricin B of *L. salivarius* M7 [23] and in two amino acids (V6I and K43I) with the presalivaricin B encoded by *L. salivarius* UCC118 [22]. However, peptides identical to SalB in *L. salivarius* P1ACE3 have been encoded by other *L. salivarius* strains [39], with no additional data for its expression, processing, and/or secretion. Thus, to confirm the functionality of the SalB encoded by *L. salivarius* P1ACE3, the IV-CFPS of the putative mature *salB* was performed, although no antimicrobial activity was detected against any of the indicators tested. This negative IV-CFPS-derived antimicrobial activity of the resulting product contrasts with the successful IV-CFPS and recording antimicrobial activity of many other class II bacteriocins previously assayed [40]. This suggest that either SalB has no antimicrobial activity against the indicators tested or that the peptide holds a different biological function. Another possibility is that SalB belongs to the group of ribosomally synthesized and post-translationally modified peptides (RiPPs) that undergo enzymatic modifications during their biosynthesis [41,42]. Nevertheless, genes encoding specific proteins for maturation and processing of SalB seem to be absent in *L. salivarius* P1ACE3.

Downstream of ORF4 in *L. salivarius* P1ACE3, two ORFs (ORF6 and ORF7) encoding the bacteriocin Abp118 (Abp118α and Abp118β) were identified (Figure 1a). The bacteriocin Abp118 was first described as produced by the human intestinal probiotic strain *L. salivarius* UCC118, and the genes encoding this bacteriocin located in a 242-Kb megaplasmid [33,43]. Abp118 is a broad-spectrum class IIb bacteriocin characterized by inhibiting bacterial pathogens such as *Listeria*, *Bacillus*, *Enterococcus,* and *Staphylococcus* species, but not closely-related LAB strains [33]. Bacteriocins similar to Abp118 have also been identified in *L. salivarius*, such as the salivaricin P produced *L. salivarius* DPC6005 isolated from the GIT of pigs [44] and salivaricin T produced by *L. salivarius* DPC6488, a neonatal human isolate [45]. Thus, in order to determine whether the observed direct antimicrobial activity of *L. salivarius* P1CEA3 could be, at least in part, explained by the production of Abp118, mature *abp118α* and *abp118β* were both used as templates for the IV-CFPS of Abp118α and Abp118β. The combination of both bacteriocins, but not the bacteriocins alone, resulted in the inhibition of different indicator strains (Figure 4).

However, by comparing the *abp118* gene cluster of *L. salivarius* UCC118 with that of *L. salivarius* P1CEA3, the absence of essential genes such as those encoding a dedicated ABC-transporter (*abpT*) and an accessory protein (*abpD*) in *L. salivarius* P1CEA3 (Figure 1a) can be observed. These proteins are known to be involved in the removal of the leader peptide of the synthesized bacteriocins and translocation of the mature bacteriocin through the cytoplasmic membrane [33,46]. Moreover, in the *abp118* gene cluster of *L. salivarius* P1ACE3 there are a number of single nucleotide deletions responsible for frameshift mutations in the hypothetical *abpK* gene encoding a histidine kinase protein (AbpK), thus generating a truncated version of this protein. Abp118 production is also known to be autoregulated by a three-component quorum-sensing system consisting of an inducer peptide (AbpIP), a histidine kinase (AbpK), and a response regulator (AbpR) [33]. Therefore, the hypothetical production of a shorter and, most likely, inactive version of AbpK might have a negative implication in the regulation of the expression of the genes encoding Abp118 in *L. salivarius* P1CEA3. The observed high sensitivity of *L. salivarius* P1CEA3 against the IV-CFPS produced Abp118 also suggest that, most likely, the *abp118α* and *abp118β* genes are not being expressed and the immunity protein AbpIM is not being produced. Moreover, the presence of Abp118 has not been detected during the purification of the antimicrobial peptides hypothetically produced and secreted by *L. salivarius* P1CEA3 (Figure 5c,d and Table 2). Therefore, it is possible that, although the *abp118* structural genes might be functional (Figure 1 and Table S2), the lack of an appropriate system for synthesis and production of the Abp118 as well as the lack of a dedicated processing and transport system for secretion of this bacteriocin are mostly preventing the production and secretion of this bacteriocin by *L. salivarius* P1ACE3.

The second bacteriocin gene cluster identified in *L. salivarius* P1CEA3 showed the presence of genes hypothetically encoding proteins involved in the biosynthesis of a novel nisin variant, termed nisin S. The nisin S gene cluster includes a gene encoding the structural bacteriocin (*nssA*), two genes involved in enzymatic modifications (*nssB* and *nssC*), one gene encoding an ABC-transporter ATP-binding protein (*nssT*), three genes encoding putative immunity proteins (*nssF*, *nssE* and *nssG*), and two genes encoding proteins regulating transcription via signal transduction by a two-component regulatory system (*nssR* and *nssK*) (Figure 2 and Table S2). The arrangement of genes, within the nisin S cluster, is similar to those from nisin A, Z, Q, and F (*nssABTCRKFEG*), although lacking the genes encoding a leader sequence specific peptidase and a specific immunity protein (equivalent to *nisP* and *nisI*, respectively, in the *nisA* gene cluster) (Figure 2). Another nisin operon missing a gene homolog to *nisP* is that from nisin O_1–4_ (Figure 2) [20]. Interestingly, nisin O_1–3_ and nisin S are the only nisin variants with a lysine in the cleavage site of the leader sequence (position-1) instead of arginine (Table S2). NisP is a protease that cleaves off the leader peptide of *nisA*, releasing the mature bacteriocin [47]. Although the NisP cutting specificity may be not as strict as suggested, some results support the importance of the arginine residue in the last position of the specific cleavage site of the *nisA* leader sequence peptide. In fact, it has been reported that, when the last four amino acids of the leader sequence of *nisA* (ASPR) are substituted by a protease recognition sequence of the enteropeptidase enterokinase (DDDK), the leader sequence cannot be cleaved by NisP [48]. Most likely, as it has been suggested for nisin O_1–4_ [20] and observed for other class I bacteriocins, such as subtilin [49] and mutacin I [50], that a protease encoded elsewhere in the genome of *L. salivarius* P1CEA3 might be necessary for cleavage of the nisin S leader sequence as well as processing and transport of nisin S (NisS) out of the producer cells.

Nisin-producing strains show self-immunity to nisin due to the coordinate expression of *nisI, nisF*, *nisE,* and *nisG*. NisI is a membrane-anchored lipoprotein that acts as a nisin-sequestering protein while NisFEG constitutes an ABC transporter that acts as a nisin exporter from the cytoplasmic membrane to the environment [51]. The absence of *nisI* homologs has been observed in operons from nisin H, J, and G and the nisin-like bacteriocin kunkecin A, as well as in *L. salivarius* P1ACE3 (Figure 2). It has also been described that the expression of *nisFEG* is sufficient to protect the producer cells against their own bacteriocins [8,19,22,25,51], although this self-protection is less efficient in the absence of an equivalent *nisI* [8]. This suggestion is further supported by this study, where purified NisS showed a certain degree of inhibition against *L. salivarius* P1CEA3 (Figure 2).

Colony MALDI-TOF MS revealed the presence, in the *L. salivarius* P1ACE3-isopropanol-derived extracts, of a peptide of 3347 Da (Figure 5a,b). The 144 Da difference between the deduced (3491 Da) and the observed (3347 Da) molecular mass of the identified peptide is compatible with eight dehydrations involved in the formation of lanthionine and 3-methyllanthionine bridges [52]. Since the conformations of the different nisin natural variants are highly conserved [19], and taking nisin A as a template, the novel nisin variant nisin S is proposed to have lanthionine/methyllanthionine rings between S3 and C7, T8 and C11, T13 and C19, T23 and C26, and S25 and C28 (Figure 6). Further experiments need to be done to confirm the proposed structure. Compared with nisin A [22], nisin S has seven amino acid differences, but it has the same size (34 amino acids) and the closest molecular weight with respect to nisin A, with only a 7 Da difference. However, in terms of amino acid sequence, the closest nisin variant to nisin S is nisin F, produced by a *Lactococcus lactis* strain [12], with a 82.35% amino acid identity with nisin S.

The purified nisin S showed a broad spectrum of antimicrobial activity against all Gram-positive indicators tested in this study, with the exception of *C. bovis* and *B. cereus* (Table 3). Nisin S was also slightly active against *L. lactis* subsp. *lactis* BB24, producer of nisin A, although cross immunity among nisin producer strains has been well documented [8,19]. Surprisingly, nisin S also showed a slight antimicrobial activity against *E. coli* DH5α, and it has also been documented that some bacteriocins, including nisin variants, can inhibit some Gram-negative bacteria when tested at high concentrations [25,53]. Nisin S was not active against *S. paratyphi* CECT 554, although the concentration used was, most likely, not high enough to inhibit this particular strain. Further studies with different Gram-negative indicators and higher concentrations of nisin S are needed in order to fully characterize the spectrum of activity of this bacteriocin. However, nisin S seems to have a similar spectrum of activity, but an overall higher antimicrobial activity than nisin A, against the bacterial indicators tested (Table 3).

Compared to all described nisin variants, nisin S has two unique amino residues at positions T20 and S25 of its core peptide (Figure 3 and Table S3). T20 is located in the hinge region of the molecule (positions 20, 21, and 22) which is thought to be crucial for the flexibility and reorientation of nisin after binding with lipid II for penetration into the target cell membrane [54]. This region contains the amino acids NMK in nisin A, and TMK in nisin S. Amino acid substitutions in the hinge region of nisin A and, specifically, the substitution N20T shows, in general, a higher antimicrobial activity as compared to nisin A, against methicillin resistant *S. aureus* (MRSA) and non-MRSA strains but a decreased antagonistic activity against *S. agalactiae* ATCC 13813 [55].

The pore-forming C-terminal domain of nisin S, which includes the D and E rings and the T25S, H27G, S29H, I30V, and V32I substitutions, is the region with more amino acid substitutions as compared with nisin A. The amino acid substitution T25S in nisin S involves the formation of a lanthionine bridge that stabilizes the E ring, instead of a 3-methyllanthionine bridge stabilizing the E ring in nisin A (Figure 6). At position 27 and within the E ring, nisin S (H27G) shows a hydrophobic residue (G) while nisin A has a polar amino acid (H), a substitution also observed in nisin P, nisin O, nisin U, nisin U2, and nisin E [18,20,21,23,56]. This substitution could confer nisin S has a stronger ability to permeate membranes and support, in part, its antimicrobial activity against Gram-negative bacteria such as *E. coli*, something documented for nisin J which has a hydrophobic residue at position 20 of its molecule [8]. Other amino acid substitutions such as S29H, I30V, and V32I, are found in other nisin variants and could affect the antimicrobial activity and inhibitory spectrum of nisin S.

The N-terminal region of nisin S is hypothetically responsible for its binding to the membrane of target strains [27]. Rings A, B, and C in nisin S are very similar to those in nisin A, with only one substitution I4Y (Figure 6). This substitution is also found in nisin G and kunkecin A (Figure 3 and Table S3). Nisin G has been reported to have a narrower antimicrobial spectrum than nisin A [22] while, conversely, kunkecin A has higher antimicrobial activity against *Bacillus subtilis*, *Enterococcus* sp., *Listeria innocua*, *Lactococcus lactis* (nisin A-producing LAB), and *Escherichia coli* [25]. Another interesting amino acid change in nisin S with respect to the lactococal nisins (A, Z, Q, and F) is the presence of histidine (H) instead of serine (S) at position 29 of the mature bacteriocin. It has been determined that some nisin-resistant strains produce a protease called nisin resistance protein (NSR), which is able to cut nisin A at the position S29, thus reducing the antimicrobial activity of the peptide [57]. The replacement of amino acid residues at this position render peptides resistant to the proteolytic activity of NSR [58].

In this study, we identified and characterized a novel nisin variant, termed nisin S, produced by *L. salivarius* P1ACE3 isolated from the GIT of pigs, with high antimicrobial activity against bacterial pathogens of interest in swine production. This is the first nisin variant isolated and produced by *Ligilactobacillus* sp. Since *L. salivarius* is considered to be a generally recognized as safe (GRAS) and qualified presumption of safety (QPS) microorganism with potential probiotic characteristics, future studies will determine its potential as a probiotic of interest in swine production and other potential biotechnological applications.

## 4. Materials and Methods

### 4.1. Isolation and Identification of Ligilactobacillus salivarius P1CEA3

*Ligilactobacillus salivarius* P1CEA3 was isolated from the gastrointestinal tract (GIT) of slaughtered pigs at a Madrid slaughterhouse in June 2021. Briefly, 0.1 g of the cecum content was homogenized with 1 mL of peptone water (Oxoid Ltd., Basingstoke, UK). The resulting homogenate was serially diluted in peptone water, and 100 μL of each dilution were plated on MRS agar (Oxoid). Then, the plates were incubated at 37 °C aerobically for 2 days. The isolates obtained were handpicked and inoculated into 250 μL aliquots of MRS broth in 96-well plates, and grown aerobically overnight at 37 °C. A microplate replicator was used to stamp 2–5 µL spots directly from the 96-well plates onto MRS 1.5% agar plates overlayed with 5 mL BHI 0.8% agar seeded with from 10^5^ to 10^6^ CFU/mL of a *P. damnosus* CECT 4797 overnight culture. Plates were incubated at 32 °C for 24 h, after which zones of inhibition surrounding the stamped isolates were measured. Strains of interest were subjected to genomic DNA extraction using the InstaGene^TM^ matrix (BioRad, Hercules, CA, USA). DNAs isolated were used as templates to amplify a variable region of the 16S rRNA gene with primers rD1 (5′ TAA GGA GGT GAT CCA GCC 3′) and fD1 (5′ AGA GTT TGA TCC TGG CTC AG 3′) (Thermo Fisher Scientific, Waltham, MA, USA) [59]. Purified PCR reactions were subjected to Sanger sequencing (Eurofins Genomics, Ebersberg, Germany), and the corresponding species identity were obtained by comparative sequence analysis (BLASTN) against available sequence data in the National Center for Biotechnology Information (NCBI) database.

### 4.2. Bacterial Strains, Media and Culture Conditions

The bacterial strains used in this study and their growth conditions are listed in Table S5. *L. salivarius* P1CEA3 and *Escherichia coli* DH5α were grown in tryptic soy broth (TSB) (Oxoid) and Luria Bertany (LB) broth (Scharlab, Barcelona, Spain), respectively. All indicator strains were grown in either brain heart infusion (BHI) broth or de Man, Rogosa and Sharpe (MRS) broth (Oxoid). The anaerobic growth of the strains was performed in anaerobic jars with an AnaeroGen 3.5 l pack (Oxoid). *E. coli* DH5α was grown with agitation (270 rpm) in an orbital shaker (Ecotron, Infors HT, Braunschweig, Germany). Agar plates were made by the addition of 1.5% (*w*/*v*) agar (Condalab, Madrid, Spain) to the liquid media.

### 4.3. Direct Antimicrobial Activity of L. salivarius P1CEA3

The direct antimicrobial activity of *L. salivarius* P1CEA3 was evaluated against different indicator strains. Briefly, 2 μL aliquots of an overnight culture of *L. salivarius* P1CEA3 were spotted onto 1.5% agar BHI plates. After an overnight culture of the plates at 37 °C, the grown cultures were overlaid with a layer of soft-agar (0.8%) selected medium seeded with an overnight culture of the indicator microorganism (ca. 10^5^ CFU/mL). After overnight incubation of the plates at 37 °C, the diameter of the halos of inhibition was measured.

### 4.4. Isolation of DNA, Whole Genome Sequencing (WGS), and Bioinformatic Analysis

Total genomic DNA was extracted from *L. salivarius* P1CEA3 by using the DNeasy Blood & Tissue Kit (Qiagen, Hilden, Germany). Purified DNA was quantified in a Qubit fluorometer (Invitrogen, Thermo Fisher Scientific) and its quality confirmed by agarose gel electrophoresis in 0.8% (*w*/*v*) agarose (Condalab) gels visualized with a ChemiDoc Imaging System (Bio-Rad). Whole genome sequencing (WGS) of the purified DNA was performed at the SeqCenter (Pittsburgh, PA, USA). Sample libraries were prepared using the Illumina DNA Prep kit and Integrated DNA Technologies (IDT) 10 bp unique dual index (UDI) indices, and sequenced on an Illumina NextSeq 2000 (Illumina, San Diego, CA, USA), producing 2 × 151 bp reads. Demultiplexing, quality control, and adapter trimming were performed with a BCL Convert v3.9.3 (Illumina). The resulting sequence reads were assembled into contigs using Unicycler v0.4.8 [60] and the assembly polishing rounds were done with Pilon (Oxford Nanopore Technologies, Oxford, UK). Bacterial species identification was confirmed by KmerFinder v.3.0.2 (https://cge.cbs.dtu.dk/services/KmerFinder/, accessed on 14 March 2022), which predicts bacterial species using a K-mer algorithm [61,62,63]. Annotation of the genome was performed with the Rapid Annotation Subsystem Technology (RAST) online server (http://rast.nmpdr.org/, accessed on 14 March 2022) [64]. For bacteriocin and ribosomally synthesized and post-translationally modified peptides (RiPPs) mining, the assembled genome was analyzed under default settings in the online webserver BAGELv.4.0 (http://bagel4.molgenrug.nl/, accessed on 14 March 2022) [65]. The SnapGene (GSL Biotech, San Diego, CA, USA) tool was used for analysis of the bacteriocin operons. BLASTp (NCBI) [66] and UniProt [67] confirmed peptide and protein sequences and the novelty of the putative bacteriocins identified.

### 4.5. In Vitro Cell-Free Protein Synthesis (IV-CFPS) of Bacteriocins and Peptides of Interest, and Evaluation of Their Antimicrobial Activity

To generate the amplicons used as templates for the in vitro cell-free protein synthesis (IV-CFPS) reactions, total genomic DNA from *L. salivarius* P1CEA3 was used for amplification by PCR of genes encoding the mature bacteriocins and peptides of interest. All forward primers contained the nucleotide sequence of the T7 promoter followed by the first 27 bases of the mature gene of interest, while all reverse primers contained the nucleotide sequence of the T7 terminator followed by the last 24 bases of the gene of interest (Table S3). Oligonucleotide primers were obtained from Thermo Fisher Scientific. PCR amplifications were performed with the Phusion Hot Start II High-Fidelity DNA Polymerase (Thermo Fisher Scientific) in 50 μL reaction mixtures containing 1 μL of purified DNA. PCR reactions were visualized by agarose gel electrophoresis in a ChemiDoc Imaging System (Bio-Rad), and quantified in a Qubit fluorometer (Invitrogen, Thermo Fisher Scientific). Purified PCR-derived amplicons were used as templates for the IV-CFPS of the bacteriocins and peptides of interest by using a PURExpress In Vitro Protein Synthesis Kit (New England Biolabs, Ipswich, MA, USA) [40]. The PCR-derived amplicons were used at a final concentration of 10 ng/µL in 25 µL reactions, maintained at 37 °C for 2 h, and then placed on ice to stop the reaction. The antimicrobial activity of the IV-CFPS reactions was evaluated by using a spot-on-agar test (SOAT) [40], by depositing 5 μL samples on the surface of petri plates overlaid with a soft-agar (0.8%) culture of the indicator microorganism (ca. 10^5^ CFU/mL). The indicator microorganisms used in the IV-CFPS reactions were *L. salivarius* PG21, *L. salivarius* P1CEA3, *P. damnosus* CECT 4797, *L. seeligeri* CECT 917, *L. monocytogenes* CECT 4032, *S. aureus* ZTA11/00117ST, *S. suis* CECT 958, *S. suis* C2969/03, and *E. coli* DH5α. The antimicrobial activity of the IV-CFPS-bacteriocins Abp118α and Abp118β was evaluated in both ways, separately and together. When tested alone, 2.5 μL of each bacteriocin was mixed with 2.5 μL of dH_2_O. When tested together, both bacteriocins were mixed at an equal ratio (2.5 μL:2.5 μL). The antimicrobial activity of the IV-CFPS-produced SalB was tested as described by the Abp118 bacteriocins alone.

### 4.6. Culture Media, Growth Conditions, and Antimicrobial Activity in Supernatants of L. salivarius P1CEA3

The antimicrobial activity of cell-free supernatants (CFS) of *L. salivarius* P1ACE3 was evaluated under different culture media and growth conditions. The producer strain was grown overnight in MRS broth at 37 °C. Then, 10 mL of MRS, BHI, and TSB broth were inoculated with a 1% and 2% inoculum of the overnight culture, and let grow at 37 °C in both aerobic and anaerobic conditions for 16 h, after which the final OD_600_ of the cultures was obtained. Similarly, 1 L of TSB broth was inoculated with 20 mL of an overnight culture of *L. salivarius* P1CEA3, and grown at 37 °C under anaerobic conditions. Then, culture samples of 10 mL were collected after 24, 48, 72, 120, and 148 h of growth. Cell-free culture supernatants (CFS) were obtained by centrifugation of cultures at 12,000× *g*, at 4 °C for 10 min, filtered through 0.22 μm pore-size syringe filters (Sartorius, Göttingen, Germany), and stored at −20 °C until further use. Then, the antimicrobial activity of the CFS was quantified by using a microtiter plate assay (MPA), as previously described [68]. For the MPA, the growth inhibition of sensitive cultures were measured spectrophotometrically at 620 nm with a FLUOstar OPTIMA (BMGLabtech, Ortenberg, Germany) plate reader. One bacteriocin unit (BU) was defined as the reciprocal of the highest dilution of the bacteriocin that caused a growth inhibition of 50% (50% of the turbidity of the control culture without bacteriocin).

### 4.7. Colony Matrix-Assisted Laser Desorption/Ionization Time of Flight Mass Spectrometry (MALDI-TOF MS) of L. salivarius P1CEA3

The colony MALDI-TOF MS analysis of *L. salivarius* P1ACE3 was performed as previously described, with slight modifications [22]. Briefly, single colonies of this strain, grown at 37 °C for 48 h on TSB agar plates, were picked and mixed with 50 μL 100% isopropanol with 0.1% (*v*/*v*) trifluoroacetic acid (TFA). The mix was vortexed three times and centrifuged at 13,000 rpm for 30 s, after which 1 μL of supernatants was mixed with 1 μL of a sinapic acid matrix (Sigma-Aldrich, St. Louis, MO, USA) in 30% acetonitrile and 0.3% TFA, and then applied directly to the MS target plate and dried under a stream of warm air. The MALDI-TOF MS analysis of samples was done on an Ultraflex workstation (Bruker Daltonics, Billerica, MA, USA) equipped with a 337 nm nitrogen laser, at the Unidad de Espectrometría de Masas (CAI Técnicas Químicas, UCM, Madrid, Spain). The mass spectrometer was calibrated with protein calibration standard I (4000–20,000 m/z) from Bruker Daltonics. The FlexControl Software v.2.4. (Bruker Daltonics) was used for sample analysis and control of method parameters. The *L. lactis* subsp. *lactis* BB24 (nisin A producer) strain, grown at 32 °C for 48 h on MRS agar plates was used as a positive control in the colony MALDI-TOF MS analysis of *L. salivarius* P1ACE3.

### 4.8. Effect of Heat, pH, and Proteases on Cell-Free Supernatants (CFS) of L. salivarius P1CEA3

The antimicrobial activity of cell-free supernatants (CFS) from *L. salivarius* P1ACE3, previously grown on TSB at 37 °C under anaerobic conditions, was evaluated after heat, pH, and protease sensitivity assays. Then, 30 µL aliquots of the CFS were heated at 37, 70, 100, and 120 °C for 10 min, for evaluation of its thermal stability, and 500 µL aliquots of the CFS adjusted to 2.0, 4.5, 7.0, and 9.5 by adding 1 M HCl or 1 M NaOH, for evaluation of its pH sensitivity. Finally, 30 µL aliquots of the CFS were mixed with proteinase K (Sigma Aldrich) to a final concentration of 20 mg/mL and maintained at 37 °C for 1 h, for determination of its protease sensitivity. After each treatment, the residual antimicrobial activity of the CFS was determined by using a SOAT, as described above, with *P. damnosus* CECT 4749 as the indicator microorganism.

### 4.9. Purification of the Bacteriocins Produced by L. salivarius P1CEA3

Bacteriocins were purified from 1 L cultures of *L. salivarius* P1ACE3, grown at 37 °C for 72 h in TSB broth under anaerobic conditions, and centrifuged at 8000 rpm for 10 min at 4 °C to obtain the corresponding cell-free supernatants (CFSs). Then, the CFSs were subjected to precipitation with ammonium sulphate, desalted by gel filtration (PD-10 columns), and further subjected to hydrophobic-interaction (Octyl-Sepharose CL-4B) chromatography, followed by two rounds of reverse-phase chromatography in an ÄKTA purifier fast protein liquid chromatography (RP-FPLC) system (GE Healthcare Life Sciences, Barcelona, Spain), as previously described [69]. For the RP-FPLC rounds of purification, the samples were initially applied into a SOURCE 15RPC ST 4.6/150 column (Cytiva, Marlborough, MA, USA) and the bacteriocins eluted with a gradient of 0% to 100% isopropanol with 0.1% (*v*/*v*) trifluoroacetic acid (TFA). The eluted fractions were monitored at 254 nm (A_254_), filtered through 0.22 μm filters (Sartorius, Göttingen, Germany), and their antimicrobial activity quantified by a microtiter plate assay (MPA), as described above, against *P. damnosus* CECT 4749 as the indicator microorganism. The fractions with the highest antimicrobial activity were then applied into a SOURCE 5RPC ST 4.6/150 column (GE Healthcare Life Sciences), and the bacteriocins eluted as described in the first round of purification. The eluted fractions with the highest antimicrobial activity were analyzed by MALDI-TOF MS at the Unidad de Espectrometría de Masas (CAI Técnicas Químicas, UCM, Madrid, Spain), as previously described. The nisin A, produced by *L. lactis* BB24, was also purified using the same described protocol.

### 4.10. Targeted Proteomics Identification Combined with Massive Peptide Analysis of the Eluted Purified Fractions with Higher Antimicrobial Activity, Derived from L. salivarius P1ACE3

Purified RP-FPLC fractions from the CFS of *L. salivarius* P1ACE3 were subjected to LC-MS analysis at the Unidad de Proteómica (CAI Técnicas Biológicas, UCM, Madrid, Spain). Briefly, peptides and proteins were reduced with 10 mM dithiothreitol (DTT) at 56 °C for 60 min, and then alkylated with 25 mM iodacetamide for 60 min. Sodium dodecyl sulfate (SDS) at 20% and 1 M tetraethylamonium bromide (TEAB) were then added, and the samples further acidified with phosphoric acid at 12%. The samples were then loaded into S-TrapTM micro columns (ProtiFI) with the S-Trap protein binding buffer (STBB) at a ratio of 6:1, and digested with 1.5 μg of recombinant trypsin (Roche Molecular Biochemicals, NJ, USA) in 50 mM tetraethylammonium bromide (TEAB) at 47 °C for 90 min. Finally, the resulting peptides were eluted, dried in a SpeedVacTM concentrator (Thermo Fisher Scientific), reconstituted at their initial volume with 2% acetonitrile (ACN) and 0.1% formic acid (FA), and quantified in a Qubit fluorometer (Invitrogen, Thermo Fisher Scientific).

Approximately 1 μg of the peptides were analyzed by liquid nano-chromatography (Vanquish Neo, Thermo Fisher Scientific) coupled to a Q-Exactive HF high-resolution mass spectrometer (Thermo Fisher Scientific). Peptides were concentrated on-line by reverse phase chromatography (RP) using an Acclaim PepMap 100 precolumn (Thermo Fisher Scientific), and then separated on a C18 Picofrit column (Thermo Fisher Scientific Easy Spray Column, PepMap RSLC C18n) with an integrated spray tip, operating at a flow rate of 250 nL/min. Then, the peptides were eluted using a gradient from 2% to 35% buffer A (0,1% AF in dH2O) for 90 min and up to 45% buffer B (0.1% AF in ACN) for an additional 10 min. A combined targeted proteomics method (data-dependent acquisition) plus an inclusion list with the m/z of fragments from the in silico digestion of the complete amino acid sequence of the peptides nisin S, Abp118α, and Abp118β was used to detect the peptides of interest in the samples. The inclusion list was obtained with the free program Skyline v.20.2 (https://skyline.gs.washington.edu, accessed on 1 July 2022). The peptides were detected in full scan MS mode with a resolution of 60,000 over a mass range m/z of 350–1800 Da. In each MS microscan, up to 15 precursors with a charge from +2 to +4 were selected depending on their intensity (threshold, 1.3 × 10^4^) with dynamic exclusion of 10 s, followed by isolation with a window width of +/−2 units of m/z, during a maximum time of 120 ms, for fragmentation by high collision dissociation (HCD) with a normalized collision energy of 20%. The MS/MS spectra were acquired with a resolution of 30,000 in positive mode.

Peptide and protein identification from MS/MS data were carried out using the Proteome Discoverer 2.4 software (Thermo Fisher Scientific) using the MASCOT v 2.6 or Sequence HT search engines and Peaks Studio v 10.5 software (Bioinformatic solution Inc., Waterloo, ON, Canada) [70]. Database searches were performed against three databases: SwissProt (562755 sequences) downloaded from Uniprot (https://www.uniprot.org, accessed on 1 July 2022), Target DB with the 3 target proteins of *Ligilactobacillus salivarius,* and a Contaminants DB (247 sequences) including common contaminants, such as keratins, BSA, and trypsin (www.matrixscience.com/help/seq_db_setup_contaminants.html, accessed on 1 July 2022). MS/MS spectra acquired from the samples were also analyzed via the Peaks Studio v.10.5 software [70] that has different tools for peptide and protein identification: database searching (NODE PEAKS), de novo sequencing (NODE DE NOVO), analysis and characterization of post-translational modifications (NODE PEAKS PTMs), and mutation detection (SPIDER), to maximize the number of peptides and proteins identified. The data acquired from the samples (raw data) were imported into the software. First, the data are filtered according to the charge, the mass of the precursors is recalibrated, and after refinement, the identification is performed using Peaks as the search engine with the search parameters, against the databases. The Target database with the three sequences of interest and the Contaminants database (247 sequences) were also used to eliminate the most common contaminating proteins. The acquired data were analyzed using the Skyline v.21.2 software to assign the precursors and transitions to the peptides selected in the targeted proteomics method to ensure the detection of the peptide and its sequence. The identification results obtained in Proteome Discoverer were incorporated as a library of spectra in skyline in order to confirm the agreement (dotp) between the transitions and the sequences (MS/MS spectra) of the peptides.

### 4.11. Antimicrobial Activity of the Purified Nisin S against Different Indicator Strains

For determination of the antimicrobial activity of the purified nisin S, produced by *L. salivarius* P1ACE3, 5 µL of the purified fraction, resulting from the second round of purification by RP-HPLC, were used on a SOAT assay, as described above, against different indicator strains. The nisin A, produced by *L. lactis* subsp. *lactis* BB24, and purified in the same way as nisin S, was also evaluated for its antimicrobial activity against different indicator strains. The concentrations of purified nisin S and nisin A were determined on a Qubit fluorometer (Invitrogen, Thermo Fisher Scientific) and adjusted, so that both preparations had the same concentration.

## Figures and Tables

**Table 1:** Direct antimicrobial activity of *L. salivarius* P1CEA3 against different indicator strains.

Indicator Strain	Activity ^a^
*Ligilactobacillus salivarius* P1CEA3	-
*Lactococcus lactis* BB24	-
*Lactococcus lactis* WA2–67	-
*Pediococcus damnosus* CECT 4797	+++
*Pediococcus pentosaceus* FBB61	+++
*Listeria seeligeri* CECT 917	++
*Listeria monocytogenes* CECT 4032	+
*Listeria innocua* CECT 910	+
*Staphylococcus aureus* ZTA11/00117ST	+
*Staphylococcus aureus* ZTA11/00310ST	+
*Streptococcus suis* CECT 958	++
*Streptococcus suis* C2969/03	++
*Escherichia coli* DH5α	+

^a^ Direct antimicrobial activity calculated as the diameter of the zone of inhibition, +: <10 mm, ++: 10–20 mm, +++: >20 mm.

**Table 2:** Peptide fragments identified by targeted proteomics combined with massive peptide analysis of nisin S, in the RP-FPLC-purified fractions from *L. salivarius* P1CEA3.

Peptide Sequence ^a^	Amino Acid Position	Theoretical MH+ (Da)	Found in Sample	Detected m/z
ITSYSL**C**TPG**C**K	[1–12]	1385.63	High	693.82
TGALMG**C**TMK	[13–22]	1069.48	High	535.24
TGAL**M**G**C**TMK	[13–22]	1085.47	High	543.24
TGAL**M**G**C**T**M**K	[13–22]	1101.47	High	551.24
TAS**C**G**C**DVHISK	[23–34]	1355.61	High	339.30

^a^ Cysteine carbamidomethylation and methionine oxidation are shown in bold. Amino acid sequence of NisS: ITSYSLCTPGCKTGALMGCTMKTASCGCHVHISK.

**Table 3:** Antimicrobial activity and spectrum of activity of the purified nisin S and nisin A, against different indicator strains.

Indicator Strain	Activity ^a^	
	Nisin S	Nisin A
*Ligilactobacillus salivarius* P1CEA3	++	+
*Ligilactobacillus salivarius* PG21	+++	++
*Lactococcus lactis* subsp. *lactis* BB24	+	-
*Pediococcus damnosus* CECT 4797	+++	+++
*Pediococcus pentosaceus* FBB61	+++	+++
*Enterococcus faecium* ER46	+++	+++
*Enterococcus faecalis* 12Ep11	+++	+++
*Listeria seeligeri* CECT 917	+++	++
*Listeria monocytogenes* CECT 4032	++	+
*Listeria innocua* CECT 910	++	+
*Staphylococcus aureus* ZTA11/00117ST	++	-
*Staphylococcus aureus* DICM10/00243	++	+
*Staphylococcus epidermidis* DICM15/00071	++	+
*Staphylococcus pseudintermedius* ICM21/02217	++	++
*Streptococcus suis* CECT 958	++	++
*Streptococcus suis* serotype 1 DICM10/01182–1C	++	++
*Streptococcus iniae* ATCC 29178	++	+
*Streptococcus agalactiae* DICM11/00863	+++	++
*Streptococcus dysgalactiae* VSE16/01903A	+++	++
*Streptococcus equi zooepidermicus* VSE16/00697	++	+
*Corynebacterium ulcerans* ICM19/00922–1B	++	+
*Corynebacterium pseudotuberculosis* Cam2	++	-
*Corynebacterium mastitidis* INDA2	++	-
*Corynebacterium bovis* AM3	-	-
*Bacillus cereus* ICM17/00252	-	-
*Trueperella pyogenes* ICM17/02091–1	++	-
*Erysipelothrix rhusiopathiae* ICM21/01900	+++	+
*Clostridium perfringens* DICM15/00067–5A	+++	++
*Escherichia coli* DH5α	+	-
*Salmonella paratyphi* CECT 554	-	-

^a^ Antimicrobial activity performed by a spot-on-agar-test (SOAT) and calculated as the diameter of the zone of inhibition, -: no inhibition, +: <5 mm, ++: 5–10 mm, +++: >10 mm.

## Data Availability

The whole genome assembly of *L. salivarius* P1CEA3 is deposited in GenBank under the accession number CP116812-CP116815. The nucleotide sequences reported in this study have the GenBank accession numbers from CP116812 to CP116815.

## Supplementary Materials

The following supporting information can be downloaded at: https://www.mdpi.com/article/10.3390/ijms24076813/s1.
