# Supplementary material for: Nisin S, a Novel Nisin Variant Produced by Ligilactobacillus salivarius P1CEA3
Source: Int J Mol Sci. 2023 Apr 6;24(7):6813. doi: 10.3390/ijms24076813 (PMC10095417; doi:10.3390/ijms24076813)
Supplement: Supplementary file 1 [file ijms-24-06813-s001.zip › ijms-2298891-supplementary.pdf]

Table S1. Growth of *L. salivarius* P1CEA3 in different media and antimicrobial activity and specific antimicrobial activity of their cell-free supernatants (CFSs), against *P. damnosus* CECT 4797 as the indicator microorganism.

Growth time (h)	Volume (ml)	Growth medium	Culture (OD_{600})	Antimicrobial activity (BU/ml)	Specific antimicrobial activity ([BU/ml]/OD_{600})
16	10	TSB	0.830	3,436	4,140
16	10	BHI	1.350	1,147	850
16	10	MRS	4.130	144	35
24	1,000	TSB	0.055	231	4,200
48	1,000	TSB	0.649	11,525	17,758
72	1,000	TSB	0.608	18,513	30,449
120	1,000	TSB	0.605	18,021	29,787
148	1,000	TSB	0.600	10,305	17,175

Table S2. Hypothetical functionality and identity of the SalB/Abp118 gene cluster from *L. salivarius* P1CEA3 and *L. salivarius* UCC118.

***L. salivarius* P1CEA3 ORF (gene)**[a]	**Size (aa)**	**Hypothetical functionality**	**Closest protein homologue from *L. salivarius* UCC118 (Abp118 producer)**	**% identity (% cover)**
ORF1	323	Thioredoxin family protein	ORF1	87 (100)
ORF2	61	Hypothetical protein	No hits	-
ORF3	87	Hypothetical protein	ORF2	98 (100)
ORF4 (*salB*)	57	Bacteriocin	ORF3 (*salB*)	98 (100)
ORF5	85	Bacteriocin	ORF5	100 (100)
ORF6 (*abp118α*)	64	Abp118α bacteriocin	ORF6 (*abp118α*)	100 (100)
ORF7 (*abp118β*)	68	Abp118β bacteriocin	ORF7 (*abp118β*)	100 (100)
ORF8 (*abpIM*)	55	AbpIM bacteriocin immunity protein	ORF8 (*abpIM*)	89 (100)
ORF9 (*abpIP*)	38	AbpIP bacteriocin inducer peptide	ORF9 (*abpIP*)	100 (100)
ORF10, 11, 12 (*abpK**)	17, 166, 75	AbpK sensory transduction histidine kinase	ORF10 (*abpK*)	79 (75)
ORF13 (*abpR*)	264	AbpR bacteriocin response regulator	ORF11 (*abpR*)	97 (100)
ORF14	79	Hypothetical protein	ORF12	92 (100)
ORF15	65	Hypothetical protein	No hits	-
ORF16	44	Hypothetical protein	No hits	-
ORF17	283	Hypothetical protein	ORF15,16	85 (100)
ORF18	88	Hypothetical protein	ORF17	95 (100)
ORF19	273	Site-specific integrase	ORF18	98 (100)
ORF20	656	Fructose-bisphosphatase class III	ORF19	99 (100)

[a] *abpK** corresponds to the truncated version of the abpK gene from *L. salivarius* UCC118.

Table S3. Amino acid sequence of natural nisin variants.

Nisin	Leader sequence	Mature peptide[a]
Nisin A	MSTKDFNLDLVSVSKKDSGASPR	ITSISLCTPGCKTGALMGCNMKTATCHCSIHVSK
Nisin Z	MSTKDFNLDLVSVSKKDSGASPR	ITSISLCTPGCKTGALMGCNMKTATCNCSIHVSK
Nisin Q	MSTKDFNLDLVSVSKTDSGASTR	ITSISLCTPGCKTGVLMGCNLKTATCNCSVHVSK
Nisin U	MNNEDFNLDLIKISKENNSGASPR	ITSKSLCTPGCKTGILMTCPLKTATCGCHFG
Nisin U2	MSTKDFNLDLVSVSKKDSGASPR	VISKSLCTPGCKTGILMTCPLKTATCGCHFG
Nisin F	MSTKDFNLDLVSVSKKDSGASPR	ITSISLCTPGCKTGALMGCNMKTATCNCSVHVSK
Salivaricin D	MSTKDFNLDLVEVSKSNTGASAR	FTSHSLCTPGCITGVLMGCHIQSIGCNVHIHISK
Nisin H	MSTNDFNLDLVSVSKSNAGASTR	FTSISMCTPGCKTGALMTCNYKTATCHCSIKVSK
Nisin O_{1-2}	MGKFDDFDLDVTKTAAQGGIEPK	YKSKSACTPGCPTGILMTCPLKTATCGCHITGK
Nisin O_3	MAKFDDFDLDVTKTAAQGGIEPK	YKSKSACTPGCPTGILMTCPLKTATCGCHITGK
Nisin O_4	MAKFDDFDLDIIEKKVRRDIIPAS	TSQHSFCTPNCLTGFLCPPKTQLTCTCKLKGQ
Nisin J	MNNQFNLKSKSANVSGSKGNNLETR	ITSKSLCTPGCKTGALQTCFAKTATCHCSGHVHTK
Nisin P	MNNEDFNLDLVTISKENNSGASPR	VTSKSLCTPGCKTGILMTCAIKTATCGCHFG
Kunkecin A	MSNFNDFNLGIKKVHSGSKKGLEPR	ITSYVLCTPGCITGRLMGCNNKTKTCHCHSSNHFSFHAR
Nisin G	MSTNDFNLDLVSVSKSNAGASTR	ITSYSLCTPGCKTGVLMACHLKTATCNCSIIVSK
Nisin E	MSNEDFNLDLVSVSKNSNSGASPR	ITSKSLCTPGCKTGALMTCPIKTATCGCHFGN
**Nisin S**	MSVNDFKLDLVKVSKESTNSNYSVK	ITSYSLCTPGCKTGALMGC**T**MKTA**S**CGCHVHISK

[a] Cysteines are shown in blue, and amino acid substitutions unique to nisin S are highlighted.

Table S4. Primers used in this study.

Primers and PCR products	Nucleotide sequence (5′- 3′) and description[a]	Amplification fragments
**Primers**		
abp118α-F	**GCGAATTAATACGACTCACTATAGGGCTTAAGTATAAGGAGGAAAAAATATG**AAACGTGGTCC TAACTGTGTAGGTAAC	abp118α
abp118α-R	AAACCCCTCCGTTTAGAGAGGGGTTATGCTAGTTATTATAAACAAGTAAGTGCTCCGCC	abp118α
abp118β-F	**GCGAATTAATACGACTCACTATAGGGCTTAAGTATAAGGAGGAAAAAATATG**AAAAATGGTTA TGGTGGTAGTGGAAAT	abp118β
abp118β-R	AAACCCCTCCGTTTAGAGAGGGGTTATGCTAGTTAAAAAATGGTTATGGTGGTAGTGGA	abp118β
salB-F	**GCGAATTAATACGACTCACTATAGGGCTTAAGTATAAGGAGGAAAAAATATG**AGAAATTCTTAT GATTATATAGATAGC	salB
salB-R	AAACCCCTCCGTTTAGAGAGGGGTTATGCTAGTTAACTACAAATATTTTGATTTGAGTG	salB
**PCR products**		
abp118α	T7 promoter and transcription terminator containing the mature sequence of the bacteriocin Abp118α	
abp118β	T7 promoter and transcription terminator containing the mature sequence of the bacteriocin Abp118β	
salB	T7 promoter and transcription terminator containing the mature sequence of the peptide SalB	

[a] T7 promoter is shown in bold; T7 transcription terminator is underlined.

Table S5. Growth medium and incubation conditions for the bacterial strains used in this study.

Strain	Origin[a]	Growth medium	Incubation conditions	Use in this study
Ligilactobacillus salivarius P1CEA3	DNBTA	TSB	37 ºC/ Anaerobiosis	Nisin S producer/ Indicator
Lactococcus lactis BB24	DNBTA	MRS	32 ºC/ Aerobiosis	Nisin A producer/ Indicator
Lactococcus lactis WA2-67	DNBTA	MRS	32 ºC/ Aerobiosis	Nisin Z producer/ Indicator
Ligilactobacillus salivarius PG21	DNBTA	MRS	37 ºC/ Anaerobiosis	Indicator
Pediococcus damnosus CECT 4797	CECT	MRS	32 ºC/ Aerobiosis	Indicator
Pediococcus pentosaceus FBB61	DNBTA	MRS	32 ºC/ Aerobiosis	Indicator
Enterococcus faecium ER46	VISAVET	MRS	37 ºC/ Aerobiosis	Indicator
Enterococcus faecalis 12Ep11	DNBTA	MRS	37 ºC/ Aerobiosis	Indicator
Listeria seeligeri CECT 917	CECT	BHI	37 ºC/ Aerobiosis	Indicator
Listeria monocytogenes CECT 4032	CECT	BHI	37 ºC/ Aerobiosis	Indicator
Listeria innocua CECT 910	CECT	BHI	37 ºC/ Aerobiosis	Indicator
Staphylococcus aureus ZTA11/00117ST	VISAVET	BHI	37 ºC/ Aerobiosis	Indicator
Staphylococcus aureus DICM10/00243	VISAVET	BHI	37 ºC/ Aerobiosis	Indicator
Staphylococcus epidermidis DICM15/00071	VISAVET	BHI	37 ºC/ Aerobiosis	Indicator
Staphylococcus pseudintermedius ICM21/02217	VISAVET	BHI	37 ºC/ Aerobiosis	Indicator
Streptococcus suis CECT 958	CECT	BHI	37 ºC/ Aerobiosis	Indicator
Streptococcus suis serotype 1 DICM10/01182-1C	VISAVET	BHI	37 ºC/ Aerobiosis	Indicator
Streptococcus iniae ATCC 29178	VISAVET	BHI	37 ºC/ Aerobiosis	Indicator
Streptococcus agalactiae DICM11/00863	VISAVET	BHI	37 ºC/ Aerobiosis	Indicator
Streptococcus dysgalactiae VSE16/01903A	VISAVET	BHI	37 ºC/ Aerobiosis	Indicator
Streptococcus equi zooepidermicus VSE16/00697	VISAVET	BHI	37 ºC/ Aerobiosis	Indicator
Corynebacterium ulcerans ICM19/00922-1B	VISAVET	BHI	37 ºC/ Aerobiosis	Indicator
Corynebacterium pseudotuberculosis Cam2	VISAVET	BHI	37 ºC/ Aerobiosis	Indicator
Corynebacterium mastitidis INDA2	VISAVET	BHI	37 ºC/ Aerobiosis	Indicator
Corynebacterium bovis AM3	VISAVET	BHI	37 ºC/ Aerobiosis	Indicator
Bacillus cereus ICM17/00252	VISAVET	BHI	37 ºC/ Aerobiosis	Indicator
Trueperella pyogenes ICM17/02091-1	VISAVET	BHI	37 ºC/ Aerobiosis	Indicator
Erysipelothrix rhusiopathiae ICM21/01900	VISAVET	BHI	37 ºC/ Aerobiosis	Indicator
Clostridium perfringens DICM15/00067-5A	VISAVET	BHI	37 ºC/ Anaerobiosis	Indicator
Escherichia coli DH5α	Thermofisher	LB	37 ºC/ Agitation	Indicator
Salmonella paratyphi CECT 554	CECT	BHI	37 ºC/ Aerobiosis	Indicator

[a] DNBTA: Departamento de Nutrición, Bromatología y Tecnología de los Alimentos, Facultad de Veterinaria, Universidad Complutense de Madrid (UCM), Madrid, (Spain). VISAVET: Centro de Vigilancia Sanitaria Veterinaria, Universidad Complutense de Madrid (UCM), Madrid, (Spain). CECT: Colección Española de Cultivos Tipo, Valencia, (Spain).

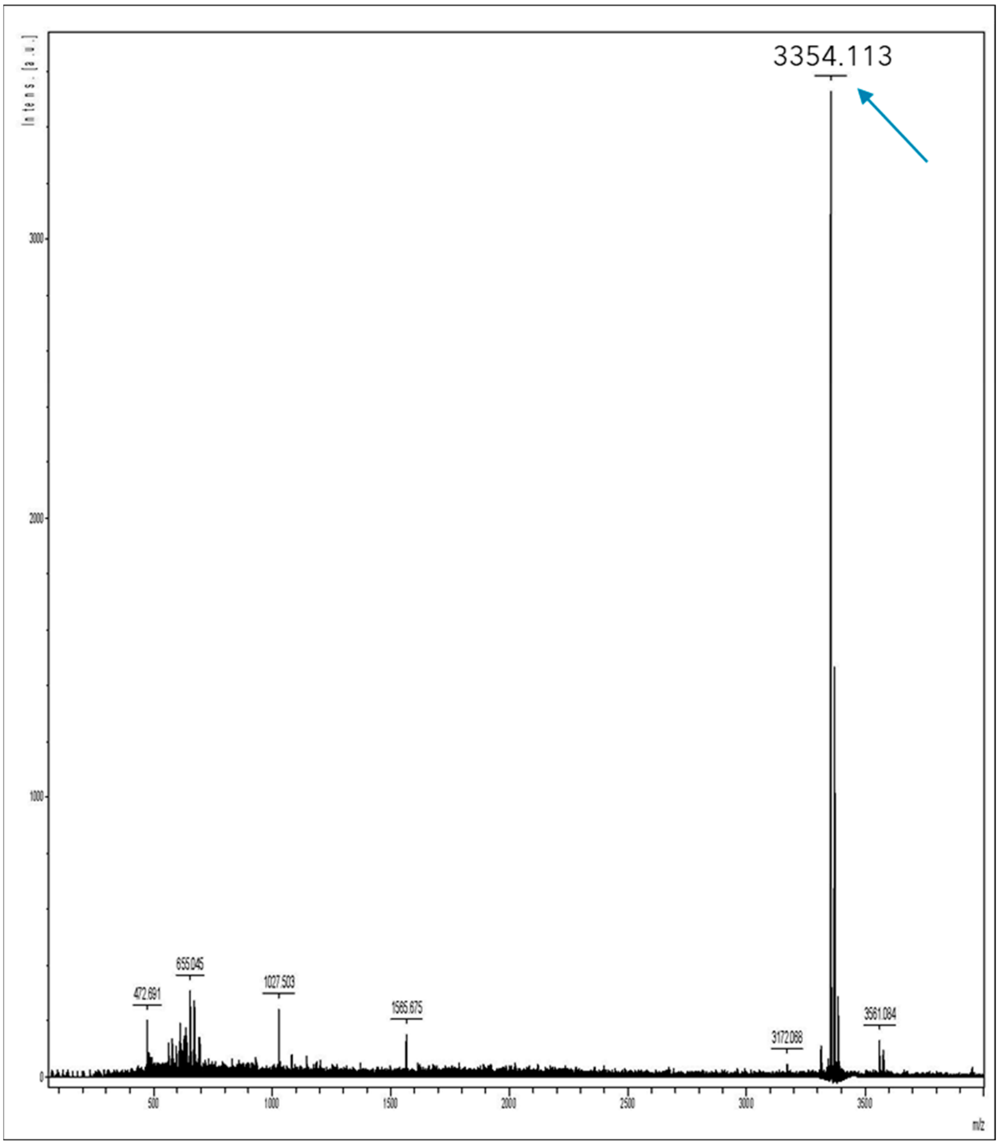

Figure S1. MALDI-TOF MS of the RP-FPLC-derived active fraction from the cell-free supernatant (CFS) from *Lactococcus lactis* subsp. *lactis* BB24 (nisin A producer).
